# SIRT3-mediated inhibition of FOS through histone H3 deacetylation prevents cardiac fibrosis and inflammation

**DOI:** 10.1038/s41392-020-0114-1

**Published:** 2020-02-28

**Authors:** Xavier Palomer, M. Silvia Román-Azcona, Javier Pizarro-Delgado, Ana Planavila, Francesc Villarroya, Brenda Valenzuela-Alcaraz, Fátima Crispi, Álvaro Sepúlveda-Martínez, Irene Miguel-Escalada, Jorge Ferrer, J. Francisco Nistal, Raquel García, Mercy M. Davidson, Emma Barroso, Manuel Vázquez-Carrera

**Affiliations:** 1 Department of Pharmacology, Toxicology and Therapeutic Chemistry, IBUB (Institut de Biomedicina de la Universitat de Barcelona), Research Institute - Pediatric Research Institute, Hospital Sant Joan de Déu, and CIBER de Diabetes y Enfermedades Metabólicas Asociadas (CIBERDEM), Faculty of Pharmacy and Food Sciences, University of Barcelona, Barcelona, Spain; 2 Department of Biochemistry and Molecular Biomedicine, IBUB and CIBER Fisiopatología de la Obesidad y Nutrición (CIBEROBN), Faculty of Biology, University of Barcelona, Barcelona, Spain; 3 aBCNatal - Barcelona Center for Maternal–Fetal and Neonatal Medicine (Hospital Clínic and Hospital Sant Joan de Deu), Institut d’Investigacions Biomèdiques August Pi i Sunyer, Universitat de Barcelona, and Center for Biomedical Research on Rare Diseases (CIBER-ER), Barcelona, Spain; 4 Genomic Programming of Beta-cells Laboratory, Institut d’Investigacions August Pi i Sunyer (IDIBAPS), and CIBERDEM, Barcelona, Spain; 5 Section of Epigenomics and Disease, Department of Medicine, and National Institute for Health Research (NIHR) Imperial Biomedical Research Centre, Imperial College London, London, UK; 6 Servicio de Cirugía Cardiovascular, Hospital Universitario Marqués de Valdecilla, Departamento de Ciencias Médicas y Quirúrgicas, Facultad de Medicina, Universidad de Cantabria, Instituto de Investigación Marqués de Valdecilla (IDIVAL), Centro de Investigación Biomédica en Red Cardiovascular (CIBERCV), Instituto de Salud Carlos III, Santander, Spain; 7 Departamento de Fisiología y Farmacología, Facultad de Medicina, Universidad de Cantabria, Instituto de Investigación Marqués de Valdecilla (IDIVAL), Santander, Spain; 8 Department of Radiation Oncology, Columbia University, New York, NY USA

**Keywords:** Cardiology, Cardiovascular diseases

## Abstract

Sirtuin 3 (SIRT3) is a deacetylase that modulates proteins that control metabolism and protects against oxidative stress. Modulation of SIRT3 activity has been proposed as a promising therapeutic target for ameliorating metabolic diseases and associated cardiac disturbances. In this study, we investigated the role of SIRT3 in inflammation and fibrosis in the heart using male mice with constitutive and systemic deletion of SIRT3 and human cardiac AC16 cells. SIRT3 knockout mice showed cardiac fibrosis and inflammation that was characterized by augmented transcriptional activity of AP-1. Consistent with this, SIRT3 overexpression in human and neonatal rat cardiomyocytes partially prevented the inflammatory and profibrotic response induced by TNF-α. Notably, these effects were associated with a decrease in the mRNA and protein levels of FOS and the DNA-binding activity of AP-1. Finally, we demonstrated that SIRT3 inhibits FOS transcription through specific histone H3 lysine K27 deacetylation at its promoter. These findings highlight an important function of SIRT3 in mediating the often intricate profibrotic and proinflammatory responses of cardiac cells through the modulation of the FOS/AP-1 pathway. Since fibrosis and inflammation are crucial in the progression of cardiac hypertrophy, heart failure, and diabetic cardiomyopathy, our results point to SIRT3 as a potential target for treating these diseases.

## Introduction

In response to diverse pathological stimuli, the heart may secrete numerous proinflammatory mediators, such as interleukin (IL)6, monocyte chemoattractant protein 1 (MCP1), and tumor necrosis factor (TNF)-α, which are under the control of the transcription factor nuclear factor-κB (NF-κB).^1,2^ These mediators locally enhance inflammation through their pleiotropic autocrine effects, which are carried out via downstream activation of NF-κB itself, activator protein-1 (AP-1) and nuclear factor of activated T-cells (NFAT), all associated with cardiovascular disease development and progression.^2–4^ For instance, TNF-α overproduction is boosted in the cardiac tissue of hypertensive rodents and human subjects with heart failure, which contributes to remodeling and functional impairment of the heart and accelerates the development of heart failure.^5^ Similarly, continuous cardiac secretion of TNF-α after transplantation favors cardiac allograft hypertrophy.^6^ In addition to cytokines and chemokines, several other factors may enhance NF-κB transcriptional activity, such as hyperglycemia, increased free fatty acid levels or reactive oxygen species (ROS).^1^

Myocardial injury caused by inflammatory processes results in left ventricular remodeling, characterized by myocyte hypertrophy, loss of functioning myocytes and interstitial fibrosis, which eventually transitions to cardiac hypertrophy and heart failure.^1,2^ Tissue fibrosis is a distinct deleterious alteration that is observed in several cardiac diseases. It occurs because of excess accumulation of extracellular matrix proteins in cardiac tissue^7^ in a process that is regulated by AP-1 and NF-κB, among others. AP-1 binds to specific promoter elements in target genes to regulate mRNA expression in response to inflammatory stimuli.^8^ In the heart, AP-1 induces the deposition of collagen and the synthesis of endothelin-1, fibronectin, and transforming growth factor-β (TGF-β).^4,7,9^ By these means, AP-1 brings about modifications to the extracellular matrix and reduces cardiac contractility, inducing fibrosis of the interstitial substance and cardiomyocyte hypertrophy, eventually leading to heart failure.^4^

Several studies have revealed multiple important links between the sirtuin family of proteins and inflammatory processes. Transgenic mice with forced overexpression of SIRT1 that were fed a Western-type diet show decreased inflammation due to downmodulation of NF-κB activity.^10^ More recently, SIRT6 has been shown to attenuate NF-κB-dependent gene expression via histone H3 deacetylation at the chromatin.^11^ Sirtuins are NAD-dependent protein deacetylases that are regarded as major regulators of cellular functions. SIRT3 is ubiquitously expressed, although it is particularly expressed in organs and tissues with a high metabolic rate capacity, including the heart.^12^ Protein targets of SIRT3 include mitochondrial enzymes that regulate energy metabolism, through which SIRT3 indirectly controls the production of ROS. Thus, SIRT3 has a protective effect against oxidative stress-dependent disorders. Recent studies have demonstrated that SIRT3 knockout mice display mitochondrial protein hyperacetylation^13^ and a significant reduction in ATP production in the heart.^14^ It is worth mentioning that SIRT3 is overexpressed in experimental models of cardiac hypertrophy, whereas SIRT3 overexpression partially blunts the cardiac hypertrophic response by regulating antioxidant gene expression and the activity of the mitogen-activated protein kinase (MAPK)/extracellular signal-regulated kinase (ERK) and AMP-activated protein kinase (AMPK) pathways^12,15,16^ and by attenuating lipid accumulation in cardiomyocytes.^17^ Of note, SIRT3 reverses palmitate-induced inflammation in cultured proximal tubular cells.^18^ In this study, we investigated the promising anti-inflammatory role of SIRT3 in the heart by examining the effects of this deacetylase on TNF-α-induced inflammation and fibrosis in cardiac cells of human origin and in SIRT3 knockout mice.

## Results

### SIRT3 knockout mice display inflammation in the heart

**Fig. 1:**
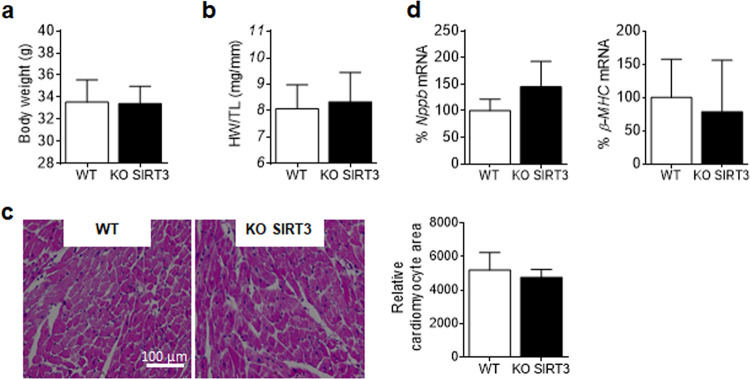
SIRT3 deletion in knockout mice does not induce cardiac hypertrophy. Body weight **a** and heart weight to tibia length (HW/TL) ratio **b** in SIRT3 knockout (KO SIRT3) mice and their wild-type (WT) littermates. **c** Representative hematoxylin and eosin stained micrographs showing transverse sections from the left ventricle myocardium and quantification of cardiomyocyte cross-sectional areas. **d** Relative quantification of *Nppb* (type B natriuretic peptide) and β-myosin heavy chain (*β-MHC*) mRNA expression. Graphs represent the quantification of adenine phosphoribosyl transferase (*Aprt*)-normalized mRNA levels expressed as a percentage of the WT samples. The data are the mean ± SD (Mann–Whitney test)

**Fig. 2:**
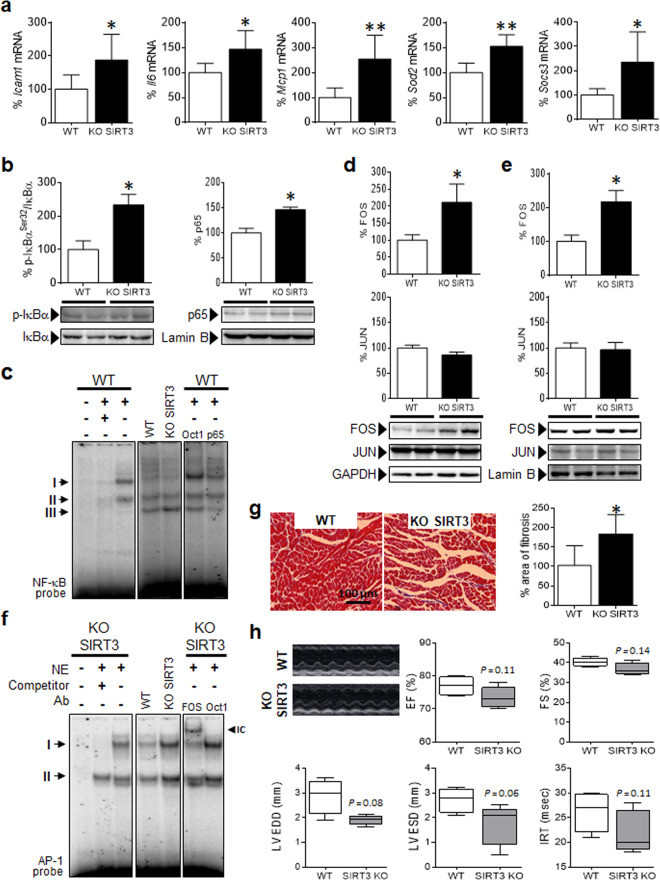
SIRT3 knockout mice display inflammation in the heart. **a** Relative quantification of *Icam1*, *Il6*, *Mcp1*, *Socs3*, and *Sod2* mRNA expression in SIRT3 knockout (KO SIRT3) and wild-type (WT) mice. **b** Western blot analysis showing the levels of p-IκBα^Ser32^/IκBα in total protein extracts and p65 in nuclear protein fractions (NE) obtained from the same samples. The data are the mean ± SD. **c** EMSA data showing NF-κB DNA-binding activity in the heart. Ab antibody; IC immunocomplex; NE nuclear extract. Western blot analysis showing the levels of FOS and JUN in cytosolic **d** and nuclear **e** protein fractions obtained from heart samples of KO SIRT3 and WT mice. The graphs represent the quantification of adenine phosphoribosyl transferase (*Aprt*)–normalized mRNA levels **a** or the normalized quantification of protein levels **b**, **d**, and **e** expressed as a percentage of the control samples ± SD. **f** EMSA data showing AP-1 DNA-binding activity in the heart. **g** Representative images of Mason’s trichrome staining and quantification of fibrosis expressed as a percentage of WT samples ± SD in the heart. **h** Representative M-mode transthoracic echocardiographic images and graphs representing evaluations of EF, FS, LV EDD, and LV ESD diameters, and IVRT. The Data are presented as the median ± interquartile range and were compared using the Mann–Whitney test. *P < 0.05, **P < 0.01, and ***P < 0.001 vs. WT

No differences were found in body weight or cardiac hypertrophic markers between SIRT3 knockout and wild-type mice (Fig. 1). However, SIRT3 knockout mice showed an increase in myocardial expression of intercellular adhesion molecule 1 (*Icam1*), *Il6*, *Mcp1*, suppressor of cytokine signaling 3 (*Socs3*), and superoxide dismutase 2 (*Sod2*) (Fig. 2a). This inflammatory profile did not arise from systemic inflammation, as plasma IL6 and MCP1 levels were not increased in knockout mice (Supplementary Fig. S1c) and was not a consequence of increased macrophage infiltration in the heart, as suggested by the absence of changes in the expression of adhesion G protein-coupled receptor E1 (*Adgre1*) and *Cd68*, which are widely recognized as macrophage-specific markers (Supplementary Fig. S1d).

Next, we aimed to elucidate whether NF-κB and AP-1 were responsible for these transcriptional changes. The hearts of SIRT3 knockout mice displayed increased phosphorylation of the NF-κB inhibitor IκBα and, in accordance with this, p65 was increased in the nucleus (Fig. 2b). To further corroborate this, we performed an EMSA. NF-κB formed three major DNA-binding complexes, but only complex III was slightly increased in the cardiac cells of SIRT3 knockout mice (Fig. 2c). In contrast, the formation of complexes I and II was slightly reduced or not modified at all.

With regard to AP-1, FOS levels but not JUN, the other partner of the AP-1 heterodimer, were increased in SIRT3 knockout mice (Fig. 2d, e). Consistent with this, AP-1 transcriptional activity was increased after SIRT3 deletion (Fig. 2f), and this was correlated with the enhanced expression of the AP-1 target gene *ATF4* (activating transcription factor 4; Supplementary Fig. S1e), a profibrotic transcription factor that controls the synthesis of type I collagen and other fibrosis-related proteins.^19^ In fact, SIRT3 knockout mice spontaneously developed myocardial fibrosis, since the collagen content in the heart was higher in knockout mice than in WT mice (Fig. 2g). Assessment of cardiac geometric parameters showed a clear tendency for smaller cavities in the presence of nonhypertrophic walls in SIRT3 knockout mice (see LV EDD and LV ESD in Fig. 2h and Supplementary Table S1). There was also a nonsignificant decrease in some radial and longitudinal parameters, such as EF, FS, MAPSE, and IRT. No differences in heart rate were observed due to loss of SIRT3, as previously reported.^20^

### SIRT3 modulates TNF-α-induced inflammation and FOS/AP-1 activity in human cardiac cells

**Fig. 3:**
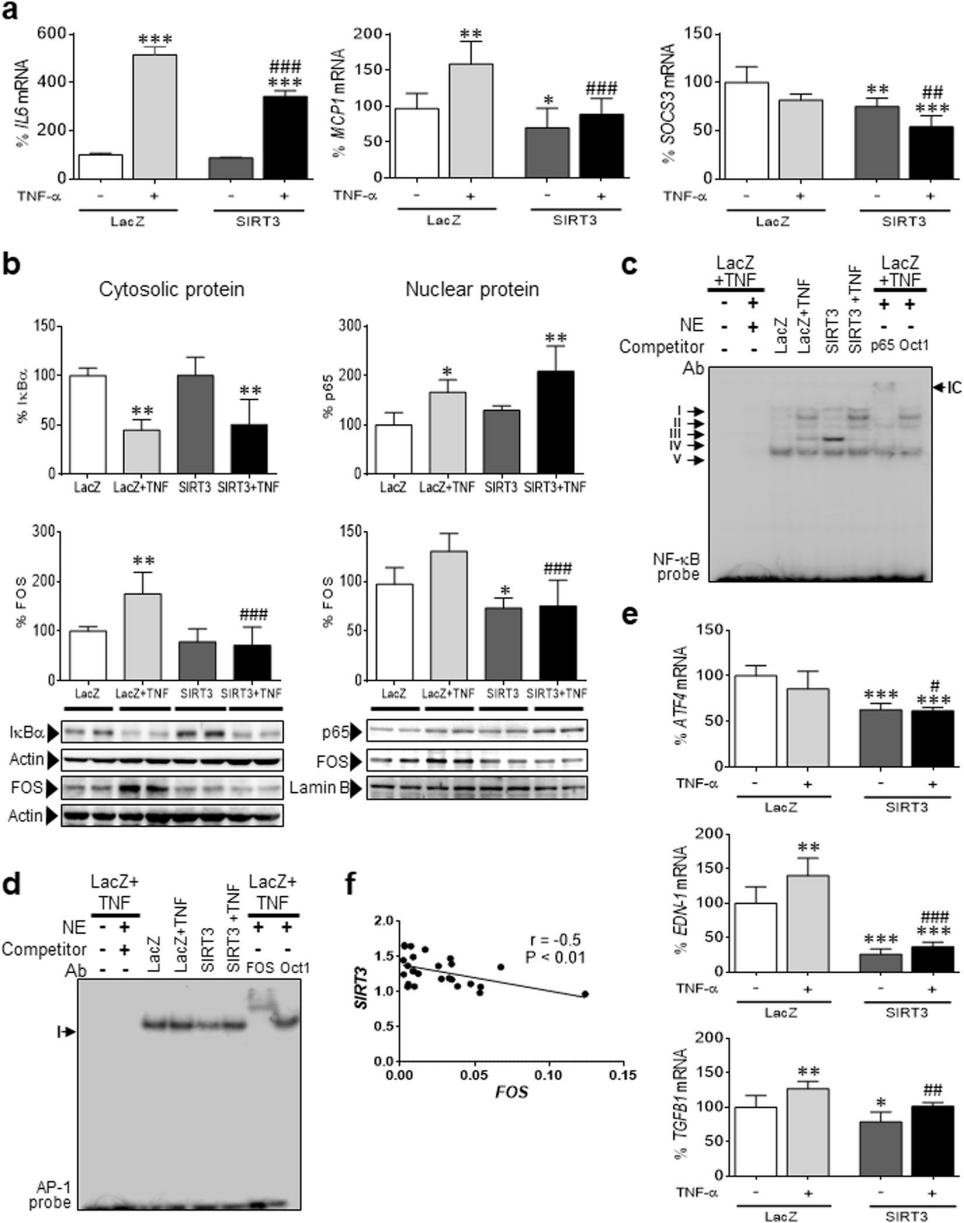
SIRT3 overexpression attenuates inflammation in human cardiac cells. **a** Relative quantification of *IL6*, *MCP1,* and *SOCS3* mRNA expression in human AC16 cardiac cells transfected with LacZ-carrying or SIRT3-carrying plasmids in the presence or absence of TNF-α (TNF, 10 ng/mL, 24 h). The graphs represent the quantification of the glyceraldehyde-3-phosphate dehydrogenase (*GAPDH*)-normalized mRNA levels, expressed as a percentage of the control samples ± SD. **b** Western blot analysis showing the levels of IκBα in total protein extracts, p65 in nuclear protein fractions, and FOS in cytosolic (CP) and nuclear (NE) protein fractions obtained from the same samples. The graphs represent the quantification of protein levels normalized to actin (total protein) or lamin B (nuclear protein), expressed as a percentage of the control samples ± SD. **c** EMSA data showing NF-κB DNA-binding activity. Ab antibody; IC immunocomplex; NE nuclear extract. **d** EMSA data showing AP-1 DNA-binding activity in the same samples depicted in panel **a**. **e** Relative quantification of *ATF4*, *EDN-1*, and *TGFB1* mRNA expression expressed as a percentage of the control samples ± SD. The data were compared by ANOVA followed by Tukey’s post hoc test. *P < 0.05, **P < 0.01, and ***P < 0.001 vs. LacZ; ^#^*P* < 0.05, ^##^*P* < 0.01, and ^###^*P* < 0.001 vs. LacZ + TNF-α. **f** Pearson correlation coefficient between SIRT3 and FOS gene expression in left ventricular myocardial tissue obtained from patients undergoing aortic valve replacement surgery. The relative transcript levels of the target genes, in arbitrary units, were used to calculate the Pearson correlation coefficient

To further confirm the function of SIRT3 in the heart, we utilized cultured cardiac cells of human origin (AC16), which were transfected with a plasmid coding for *SIRT3* (Supplementary Fig. S2a–c). In the absence of a proinflammatory stimulus, SIRT3 overexpression significantly downregulated the mRNA expression of *MCP1* and *SOCS3* (Fig. 3a). Interestingly, the predicted anti-inflammatory effect of SIRT3 was even more pronounced when a proinflammatory stimulus was added to the cells, since it partially but significantly prevented the increase in *IL6* and *MCP1* expression induced by TNF-α. Similar results were obtained with SIRT3-overexpressing cells coincubated with palmitate (Supplementary Fig. S3), a saturated free fatty acid that is responsible for the activation of inflammation in the heart.^21^ As previously reported,^22^ TNF-α stimulated the phosphorylation-induced proteasomal degradation of IκBα and subsequent p65/NF-κB nuclear translocation and activation (Fig. 3b). However, SIRT3 overexpression did not prevent or reduce the effects of TNF-α on these proteins or on the transcriptional activity of NF-κB (Fig. 3b, c). Regarding AP-1, TNF-α increased the protein levels of FOS (Fig. 3b) but not JUN (Supplementary Fig. S4); this increase was prevented by SIRT3 overexpression. Assessment of the transcriptional activity of AP-1 by EMSA demonstrated that SIRT3 overexpression downregulated its DNA-binding activity (Fig. 3d), and accordingly, the mRNA expression of *ATF4*, *EDN-1* (endothelin 1), and *TGFB1* (TGF-β) was attenuated (Fig. 3e).

We next explored this phenomenon in left ventricular intraoperative biopsies of patients with severe aortic valve stenosis who underwent aortic valve replacement surgery. Of note, the relative expression of SIRT3 negatively correlated with that of FOS (Fig. 3f, Pearson correlation coefficient *r* = −0.50), a finding that matched our previous in vitro results.

### SIRT3 also modulates inflammation and FOS levels in neonatal rat cardiomyocytes

**Fig. 4:**
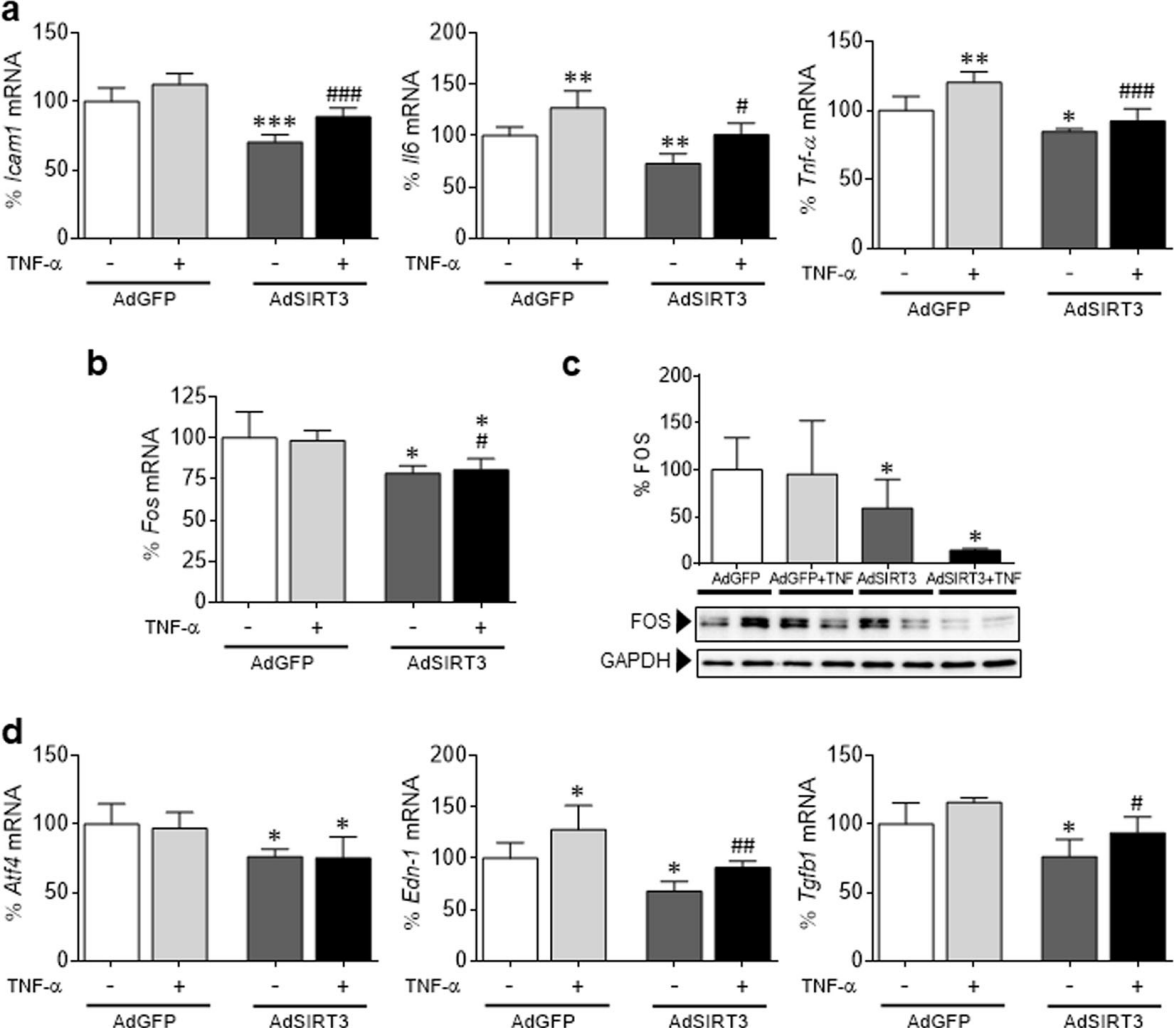
SIRT3 modulates inflammation and FOS levels in neonatal rat cardiomyocytes. Relative quantification of *Icam1*, *Il6*, *Tnf-α* **a**, *Fos* **b**, *Atf4*, *Edn-1*, and *Tgfb1* **d** mRNA expression in neonatal rat cardiomyocytes overexpressing Sirt3 (AdSIRT3) or GFP control vector (AdGFP; 30 IFU per cell, for 48 h) in the presence or absence of TNF-α (TNF, 10 ng/mL, 24 h). The graphs represent the quantification of the *Gapdh*–normalized mRNA levels expressed as a percentage of the control samples ± SD. **c** Western blot analysis showing FOS protein levels in total protein extracts obtained from the same samples depicted in panel **a**. The graph represents the quantification of protein levels normalized to GAPDH expressed as a percentage of the control samples ± SD. The data were compared by ANOVA followed by Tukey’s post hoc test. *P < 0.05, **P < 0.01, and ***P < 0.001 vs. AdGFP; ^#^*P* < 0.05, ^##^*P* < 0.01, and ^###^*P* < 0.001 vs. AdGFP + TNF-α

Transduction of neonatal rat cardiomyocytes with a recombinant adenovirus expressing murine SIRT3 cDNA (AdSIRT3) yielded a substantial increase in gene expression and protein accumulation (Supplementary Fig. S5a, b). SIRT3 overexpression significantly downregulated the mRNA expression of *Icam1*, *Il6*, and *TNF-α*, particularly in TNF-α-treated cells (Fig. 4a). This anti-inflammatory effect of SIRT3 correlated with an attenuation of *Fos* mRNA and protein levels (Fig. 4b, c) and the downregulation of the expression of the AP-1 target genes *Atf4*, *Edn-1*, and *Tgfb1* (Fig. 4d). No statistically significant changes were found in p65 protein levels in these cells (Supplementary Fig. S5b).

### SIRT3 attenuates FOS/AP-1 signaling via histone H3 deacetylation in chromatin

**Fig. 5:**
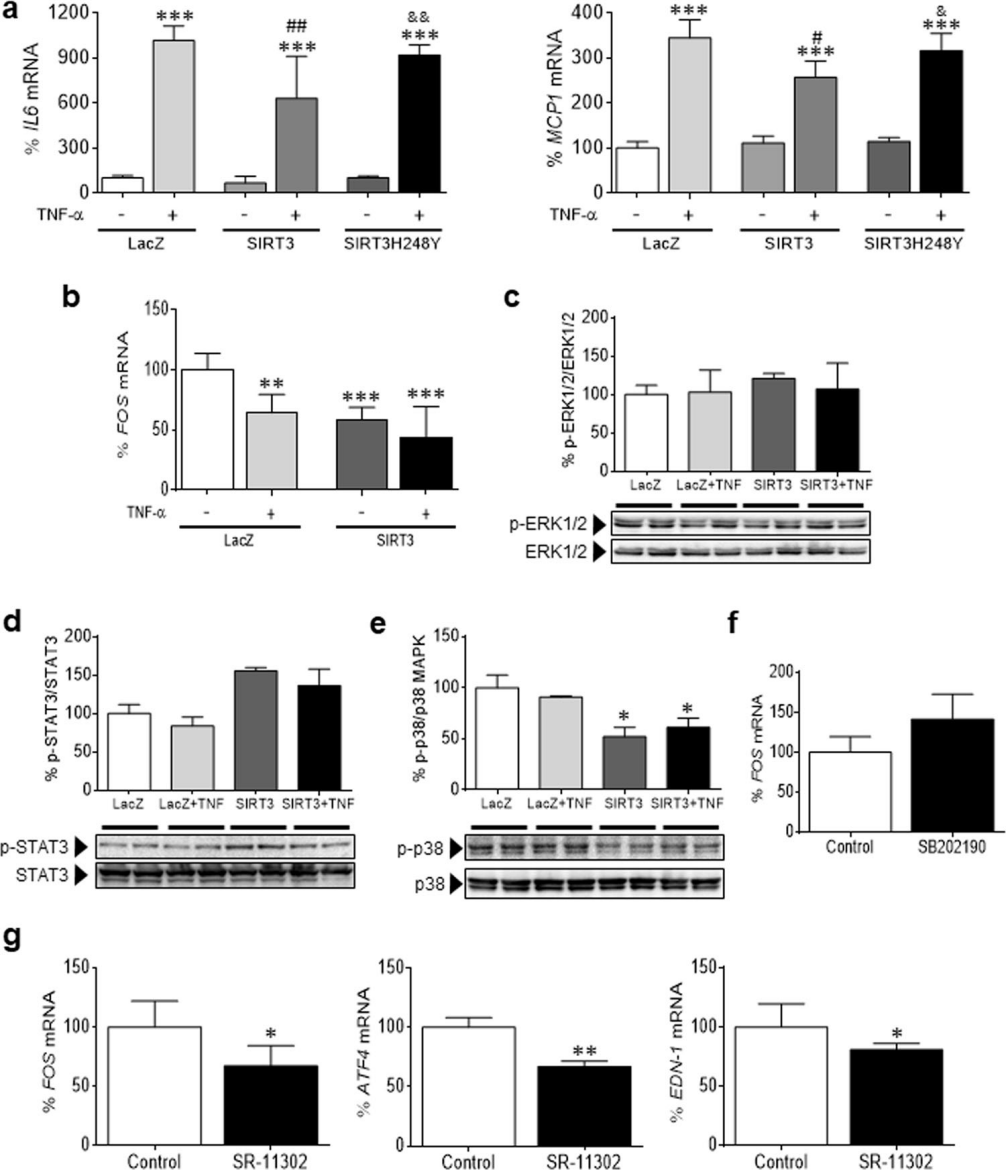
FOS is not transcriptionally regulated by STAT3 or MAPK. **a** Relative quantification of *IL6* and *MCP1* mRNA expression in human AC16 cardiac cells transfected with LacZ-carrying, SIRT3-carrying, or SIRT3H248Y-carrying plasmids in the presence or absence of TNF-α. **b** Relative quantification of FOS mRNA expression in human AC16 cardiac cells transfected with LacZ-carrying or SIRT3-carrying plasmids in the presence or absence of TNF-α. Western blot analysis showing the protein levels of phosphorylated ERK1/2^Thr202/Tyr204^/ERK1/2 **c**, phosphorylated STAT3^Ser727^/STAT3 **d**, and phosphorylated p38^Thr180/Tyr182^/p38 MAPK **e** in total protein extracts obtained from human AC16 cardiac cells transfected with LacZ-carrying or SIRT3-carrying plasmids in the presence or absence of TNF-α (TNF, 10 ng/mL, 24 h). **f** Relative quantification of *FOS* mRNA levels in human AC16 cardiac cells incubated in the presence or absence of the p38 MAPK inhibitor SB202190 (10 μmol/mL, 24 h). The graphs represent the quantification of protein levels normalized to actin and are expressed as a percentage of the control samples ± SD. **g** Relative quantification of *FOS*, *ATF4*, and *EDN-1* mRNA levels in human AC16 cardiac cells incubated in the presence or absence of the AP-1 inhibitor SR-11302 (10 μmol/L, 24 h). The graphs represent the quantification of the *GAPDH-*normalized mRNA levels **a**, **b**, **f**, and **g** or the quantification of protein levels normalized to GAPDH **c**–**e**, expressed as a percentage of the control samples ± SD. Comparisons were performed by ANOVA followed by Tukey’s posttest (panels **a**–**e**) or unpaired two-tailed Student’s *t*-test (panels **f** and **g**). **a**–**e** *P < 0.05, **P < 0.01, and ***P < 0.001 vs. LacZ; ^#^*P* < 0.05, ^##^*P* < 0.01, and ^###^*P* < 0.001 vs. LacZ + TNF-α; ^┼^*P* < 0.05, ^┼┼^*P* < 0.01, and ^┼┼┼^*P* < 0.001 vs. SIRT3; ^&^*P* < 0.05, ^&&^*P* < 0.01, and ^&&&^*P* < 0.001 vs. SIRT3 + TNF-α. **f** and **g** *P < 0.05, **P < 0.01, and ***P < 0.001 vs. Control

We next investigated the potential mechanisms by which SIRT3 regulates FOS levels. First, we examined overall acetylation levels in total and nuclear protein extracts, and no statistically significant changes were observed despite the increase in SIRT3 levels (Supplementary Fig. S2d). The involvement of the deacetylase activity of SIRT3 in its anti-inflammatory effect was demonstrated by transfecting cells with a plasmid coding for a deacetylase inactive mutant of SIRT3 (SIRT3H248Y), which did not abrogate the TNF-α-induced expression of *IL6* and *MCP1* in AC16 cells (Fig. 5a).

Since SIRT3 overexpression correlated with a decline in *FOS* gene expression (Fig. 5b), we focused on transcriptional mechanisms. The *FOS* gene promoter contains diverse regulatory elements.^23^ A major element is the v-sis-inducible element (SIE), which binds the phosphorylated STAT3 (signal transducer and activator of transcription 3) transcription factor upon cytokine treatment to participate in *FOS* promoter activation. Another key element is the serum response element (SRE); when the protein complex assembled on the SRE is phosphorylated by ERK1/2 or p38 MAPK, *FOS* transcription is induced. Western blot analysis revealed that neither STAT3 nor ERK1/2 were responsible for the effects of SIRT3 on FOS expression levels (Fig. 5c, d). In contrast, p38 MAPK activity was evidently diminished by SIRT3 (Fig. 5e). However, treatment with the p38 MAPK inhibitor SB202190 did not reduce the expression of the *FOS* gene, but rather had the opposite effect, thus ruling out the transcriptional control of the latter by this kinase (Fig. 5f). The *FOS* promoter also contains an AP-1 consensus binding site that may positively regulate its transcription.^24^ Specific pharmacological inhibition of AP-1 activity by SR-11302 significantly downregulated the transcription of *FOS* and the AP-1-target genes *ATF4* and *EDN-1* (Fig. 5g), suggesting that the SIRT3-mediated effects were due, at least in part, to the attenuation of AP-1 activity.

**Fig. 6:**
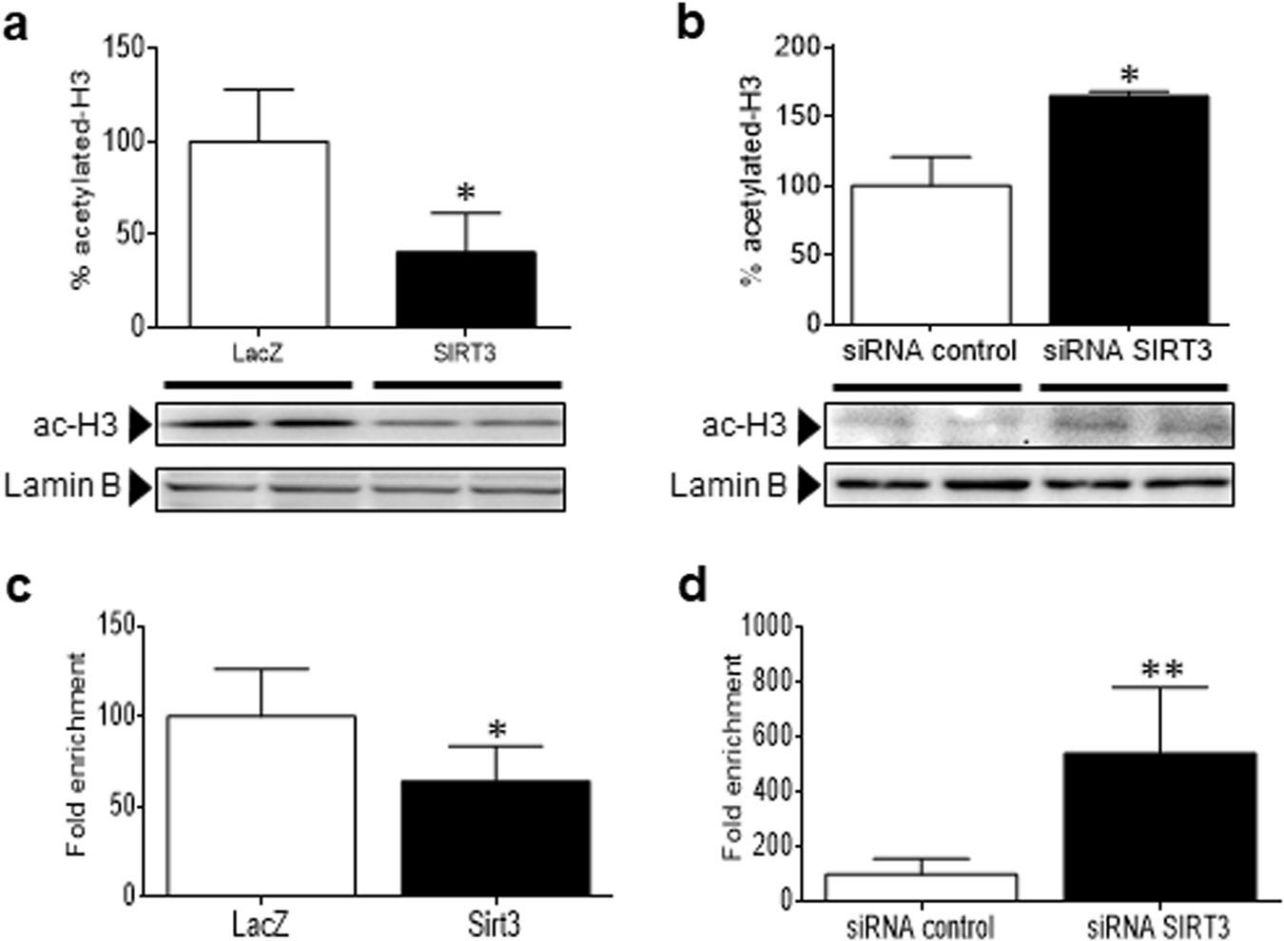
SIRT3 attenuates FOS/AP-1 signaling via H3K27 deacetylation at the chromatin. Western blot analysis showing the protein levels of acetylated histone H3 in nuclear protein extracts obtained from human AC16 cardiac cells transfected with **a** LacZ-carrying or SIRT3-carrying plasmids or **b** scrambled siRNA (siRNA control) or SIRT3 siRNA. The graphs represent the quantification of protein levels normalized to lamin B expressed as a percentage of the control samples ± SD. ChIP assay for the determination of H3K27 acetylation levels at the FOS promoter in human AC16 cardiac cells transfected with LacZ or SIRT3-carrying plasmid **c**, scrambled siRNA (siRNA control) or SIRT3 siRNA **d**. The data are the mean ± SD, and comparisons were performed by unpaired two-tailed Student’s *t*-test. *P < 0.05, **P < 0.01, and ***P < 0.001 vs. LacZ **a** and **c** or siRNA control **b** and **d**

On the other hand, previous reports indicate that nuclear sirtuins (SIRT1, SIRT2, and SIRT6) deacetylate H3 and H4 histones in chromatin in a gene-specific manner.^11,25,26^ In the framework of gene expression, histone H3 acetylation at specific lysine residues (H3K56, H3K14, H3K9, and H3K27) is associated with actively transcribed genes, while deacetylation correlates with repression. For this reason, we examined the potential role of this epigenetic mechanism. As shown in Fig. 6a, forced SIRT3 expression reduced overall histone H3 acetylation, whereas its knockdown with small interfering RNA (siRNA) yielded an increase in H3 acetylation (Fig. 6b). Histone H4 acetylation was not affected under any of these conditions (Supplementary Fig. S6). A chromatin immunoprecipitation (ChIP) assay was performed to examine whether SIRT3 is required for H3 deacetylation at the promoter of the FOS gene. This technique demonstrated that the anti-H3K27 acetylated (H3K27ac) antibody but not the mock control effectively coimmunoprecipitated H3K27ac and the FOS promoter under basal conditions. Importantly, ChIP assays revealed that H3K27 acetylation at the FOS promoter was significantly reduced following SIRT3 overexpression (Fig. 6c), which was consistent with its transcriptional repression. In contrast, in cells depleted of SIRT3 (Fig. 6d) by means of siRNA-mediated gene silencing, H3K27 was hyperacetylated at this promoter.

## Discussion

Metabolic disorders including obesity and type 2 diabetes may induce structural and functional alterations in the heart muscle that lead to compromised myocardial function, cardiac hypertrophy and, ultimately, heart failure, which is the foremost cause of death in these patients.^27^ Several, but not all, studies have reported that mice with reduced SIRT3 expression develop cardiac hypertrophy, interstitial fibrosis, and contractile dysfunction.^15,16,20,28–30^ The results presented herein reveal that SIRT3 knockout mice exhibited cardiac fibrosis and inflammation, although we did not observe cardiac hypertrophy or cardiac dysfunction, as inferred from echocardiographic data, unlike what was previously reported with the same mouse strain.^15,30^ However, our data conform to other previously published results in which cardiac function was preserved in SIRT3 knockout mice despite spontaneously developing massive cardiac fibrosis.^31–33^ We do not know the reason for the discrepancies regarding cardiac morphology and function among these studies, although the age of the mice might be an important issue.

In this study, we found that overexpression of SIRT3 but not its inactive mutant (SIRT3H248Y) in cardiac cells of human origin and in neonatal rat cardiomyocytes exerted an anti-inflammatory effect, which was even more evident in the presence of proinflammatory stimuli. Further reinforcing these data, suppression of SIRT3 increased the transcription of inflammatory genes in the hearts of knockout mice. Myocardial fibrosis was also elevated in cardiac tissue of SIRT3 knockout mice, as reported in other studies,^28,31^ and consistent with this, the expression of *TGF-β, ATF4*, and *EDN-1* was inhibited by SIRT3 overexpression in AC16 cells and in neonatal rat cardiomyocytes. This effect of SIRT3 was mostly mediated by its inhibitory effect on the FOS subunit of AP-1. In the heart, AP-1 decreases contractility and induces cardiomyocyte hypertrophy, inflammation, and fibrosis, which eventually result in heart failure.^4^ In fact, it is widely known that the FOS/AP-1 pathway induces the formation of endothelin-1, fibronectin, and TGF-β and the deposition of collagen, favoring the emergence of cardiac fibrosis.^4,7,9^ Overall, our results support the idea that AP-1 plays a crucial role in controlling inflammation and fibrosis in the heart and are consistent with the aforementioned data indicating that, in addition to NF-κB, AP-1 and other transcription factors also control cardiac cytokine and chemokine mRNA expression.^21^

An intriguing issue regarding the results reported herein is that the effects of SIRT3 spread to the nucleus, since there is still some controversy surrounding its intracellular localization. Until recently, SIRT3 activity had been regarded as being exclusively localized in the mitochondria, but there is now evidence that it is also found in the cytoplasm and the nucleus.^15,34–36^ Indeed, nuclear substrates of SIRT3 activity have already been recognized, including the H3 and H4 histones and the transcription factor FOXO3a.^15,17,37^ Now, we report for the first time that SIRT3 modulates another nuclear transcription factor, FOS/AP-1. In particular, we observed changes in FOS levels in both the nucleus and the cytoplasm; hence, SIRT3 might perform genomic (i.e., inflammation) and nongenomic^38^ actions through its effect on FOS protein levels. As previously reported in palmitate-treated pancreatic cells,^39^ nuclear and cytoplasmic SIRT3 protected cardiac cells from TNF-α-induced inflammation, despite being less abundant than mitochondrial SIRT3. Bearing in mind that other sirtuins (i.e., SIRT1, 6, and 7) are more abundant in the nucleus, and it is feasible that SIRT3 only acts on a small and selective subset of proteins and even histone residues,^34^ thus accounting for the lack of changes in the overall acetylation pattern in our study. Another possibility is that SIRT3 has a crucial function in the nucleus but is quickly and negatively regulated through its physical nuclear exclusion.^34^ Last but not least, we demonstrate that SIRT3 is located in the nucleus of cardiac cells and is recruited to the FOS promoter to deacetylate H3K27, thus hindering the accessibility of chromatin to DNA-binding factors and repressing transcription in a gene-specific manner.

Our findings show that SIRT3, by means of its action on FOS/AP-1, plays a key role in modulating fibrosis and the inflammatory response in cardiomyocytes. This is important because FOS expression is known to be increased during cardiac hypertrophy and heart failure. Since fibrosis and inflammation are crucial during the development of these cardiomyopathies, our data suggest that SIRT3 may be useful for treating these conditions. SIRT3 is also involved in the pathophysiology of obesity and type 2 diabetes.^39,40^ Interestingly, *FOS* transcription is increased as a result of insulin shortage in the heart^4^ and in adipose tissue of diabetic rats,^41^ while a frequently recognized polymorphism that decreases SIRT3 activity is linked with metabolic syndrome.^42^ Likewise, metformin, in addition to promoting glucose uptake, displays anti-inflammatory effects that are explained, at least in part, by the stimulation of SIRT3.^40^ Metabolic diseases contribute to the progression of cardiac hypertrophy and heart failure, thereby adding pharmacological value to SIRT3 and highlighting a new alternative approach for targeting insulin resistance-related diseases. In conclusion, our findings have considerable consequences not only for the treatment and management of heart failure but also for metabolic diseases characterized by chronic low-grade inflammation.

## Materials and methods

### Reagents and antibodies

[γ-^32^P]-ATP was purchased from PerkinElmer (Waltham, MA, USA), bovine serum albumin (BSA) was purchased from Calbiochem (Darmstadt, Germany), and SR-11302 was purchased from Tocris Bioscience (Bristol, United Kingdom). All other reagents were purchased from Sigma-Aldrich Corporation (St. Louis, MO, USA).

Antibodies against acetylated-lysine (#9441), total (#9102), and phosphorylated (Thr202-Tyr204, #9101) ERK1/2 MAPK, phosphorylated IκBα (Ser32, #2859), total (#9212) and phosphorylated (Thr180-Tyr182, #9211) p38 MAPK, SIRT3 (#5490) and phosphorylated (Ser727, #9134) STAT3 were obtained from Cell Signaling Technology (Danvers, MA, USA). Antibodies against FOS (sc-253), total IκBα (sc-371), JUN (sc-44), Lamin B (sc-6216), OCT1 (sc-232), p65 (sc-109), and total STAT3 (sc-482) were purchased from Santa Cruz Biotechnology, Inc. (Heidelberg, Germany), acetylated histone 4 (H4) was purchased from R&D Systems (AF5215, Minneapolis, MN, USA), the actin antibody was purchased from Sigma-Aldrich Co. LLC. (A5441), and acetylated-histone 3 (H3, #06-599) and GAPDH antibodies were from Millipore (MAB374, Saint Charles, MO, USA).

### Cell culture and transfection

The human AC16 cell line was grown and treated as previously described.^21,43^ For overexpression analyses, AC16 cells were transfected for 48 h with Lipofectamine 2000 according to the manufacturer’s instructions (Life Technologies, Barcelona, Spain) with the pcDNA4-myc-HisA-SIRT3 plasmid (Addgene #24924), which corresponded to the human full-length *SIRT3* gene, the pcDNA4-myc-HisA-H248Y-SIRT3 plasmid (Addgene #24917), which coded for a deacetylase-inactive SIRT3 mutant,^14^ and the corresponding LacZ-carrying plasmid as a control. SiRNA-mediated *SIRT3* gene silencing was performed by transfecting AC16 cells with human SIRT3 siRNA (Santa Cruz Biotechnology, Inc.), using scrambled siRNA as a control.

Neonatal rat cardiomyocytes were isolated and cultured as described by Palomer et al. ^44^ Neonatal rat cardiomyocytes were transduced with a recombinant adenovirus expressing murine Sirt3 cDNA (AdSirt3) or an AdCMV-GFP control vector (AdGFP) at 30 infectious units (IFU)/cell for 48 h in serum-free medium.

### Mouse cardiac sample preparation

Male SIRT3 knockout mice (B6; 129S5-SIRT3Gt(neo)218Lex) were purchased from the Mutant Mouse Regional Resource Center. Mice lacking SIRT3 (Supplementary Fig. S1a, b) were viable, fertile, and displayed a normal life span. The mice were housed under standard light–dark cycle (12-h light/dark cycle) and temperature (21 ± 1 °C) conditions, and food and water were provided ad libitum. Ventricular tissue was obtained from 7- to 9-month-old male mice.

All animal experiments were approved by the Institutional Animal Care and Use Bioethics Committee at the University of Barcelona, as stated in Law 5/21 July 1995 passed by the Generalitat de Catalunya, and conformed to the Guide for the Care and Use of Laboratory Animals published by The National Academies Press (US National Institutes of Health, 8th edition, 2011).

### Transthoracic echocardiography of mice

The mice were anesthetized using a combination of isoflurane (5%) and oxygen (2 L/min), which was subsequently reduced to 1.5% isoflurane to maintain the heart rate in the range of 350–450 beats/min. Transthoracic echocardiography was performed from a longitudinal parasternal plane using a Vivid Q (GE Healthcare, Norway) echocardiograph. Off-line analysis of the acquired images was used to determine ejection fraction (EF), shortening fraction (SF), septal and left ventricular free wall thicknesses, mitral annular plane systolic excursion (MAPSE), and end diastolic and end systolic diameters by M-mode. Isovolumetric relaxation time (IRT) and early transvalvular filling (*E*) and early diastolic annular peak (*E*′) velocities were measured via pulsed wave conventional and tissue Doppler, respectively.

### Histology

Hearts were fixed in 4% buffered paraformaldehyde and paraffin-embedded for subsequent hematoxylin and eosin or Masson’s trichrome staining. Transverse myocardial sections (5 μm thick) were visualized using an Olympus BX41 microscope (Olympus Iberia, Barcelona, Spain), and digital images were obtained at ×200 magnification. The cardiomyocyte cross-sectional area was analyzed in two randomly chosen sections per mouse using ImageJ software (National Institutes of Health, USA). The degree of fibrosis was determined in two randomly chosen frames from Masson’s trichrome-stained sections using ImageJ software. The percentage of interstitial fibrotic areas was calculated as the fraction of the light-blue-stained area × 100%.

### RNA preparation and analysis

RNA was isolated with Ultraspec reagent and subsequently cleaned (NucleoSpin RNA; Macherey-Nagel, Düren, Germany). Relative levels of specific mRNAs were quantified by real-time RT-PCR as previously described.^45^ The sequences of the primers used for amplification are shown in Supplementary Table S2.

### Immunoblot analysis and coimmunoprecipitation studies

Isolation of total protein extracts and cytosolic and nuclear protein fractions from AC16 cardiac cells or frozen tissue slides was performed as previously described.^45^ For immunoblotting, protein fractions were resolved by 10% SDS–PAGE and transferred to polyvinylidene difluoride membranes. Proteins were identified by using several antibodies and a chemiluminescence kit (PerkinElmer, Waltham, MA, USA).

For coimmunoprecipitation studies, FOS antibody was first bound to Protein A/G Plus agarose beads by covalent crosslinking with dimethyl pimelimidate and following a standard method. Next, cell nuclear extracts (20 μg) were diluted with dilution buffer (10 mmol/L PBS, 50 mmol/L KCl, 0.05 mmol/L EDTA, 2.5 mmol/L MgCl_2_, 8.5% glycerol, 1 mmol/L dithiothreitol, 0.1% Triton X-100, BSA 2%, and 1 mg/mL nonfat milk) and incubated on a rocker platform for 18 h at 4 °C with 100 μL Protein A/G Plus agarose beads containing 2 μg of bound FOS antibody. The agarose beads were centrifuged and washed with PBS. After centrifugation, the protein was eluted, and the supernatant was subjected to electrophoresis and immunoblotting with the anti-acetylated-lysine antibody.

### Electrophoretic mobility shift assay (EMSA)

EMSAs were carried out using double-stranded oligonucleotides for the consensus binding sites of AP-1 and NF-κB (Santa Cruz Biotechnology) as previously reported.^45^

### Chromatin immunoprecipitation

Approximately three million AC16 cells were transfected, as described above, with SIRT3-carrying or LacZ-carrying plasmids and SIRT3 siRNA or scrambled siRNA and fixed in 1% paraformaldehyde. Two independent replicates of fixed cells per treatment were pooled and sonicated to an average length of 200–1000 bp using a Bioruptor sonicator (Diagenode, Liège, Belgium). Sonication profiles were verified by using a TapeStation instrument (Agilent Genomics, Santa Clara, CA, USA). Chromatin was precleared with preblocked Dynabeads Protein G (Thermo Fisher Scientific, Waltham, MA, USA) for 1 h at 4 °C and immunoprecipitated with anti-H3K27ac (Abcam, ab4729) or anti-rabbit IgG (mock samples; Cell Signaling, #2729) overnight at 4 °C. Immune complexes were retrieved using preblocked Dynabeads Protein G (Thermo Fisher Scientific) for 2 h at 4 °C. The beads were subsequently washed and eluted prior to DNA purification using phenol:chloroform extraction. ChIP and input DNA were quantified.

Real-time quantitative PCR was used to assess specific enrichment at positive and negative genomic sequences where the protein of interest is known to be bound. The enrichment of target genes was calculated using amylase (Amy2A prom) and nanog (Nanog TSS) promoters as negative controls, as well as IgG as a control for nonspecific genomic DNA binding. Commercial SimpleChIP Human AFM Intron 1 Primers (Cell Signaling) were also used as negative control primers. Real-time q-PCR was performed in triplicate for each IP reaction using the following primer sets: Amy2A prom forward (Fwd) 5′-TGCTGCCAGAACCTAAGAAAA-3′; Amy2A prom reverse (Rev) 5′-TTGAGGGCAAACTGTTTATTCA-3′; Nanog TSS Fwd 5’-AAAGCTTGCCTTGCTTTGAA-3’; Nanog TSS Rev 5’-AGTCTCCGTGTGAGGCATCT-3’; Fos prom Fwd 5’-GAGCAGTTCCCGTCAATCC-3’; and Fos prom Rev 5’-GCATTTCGCAGTTCCTGTCT-3’. ChIP-qPCR data were normalized by using the fold enrichment method.

### Human samples

This study was carried out using left ventricular myocardial intraoperative biopsies obtained from a cohort of 26 male patients (Supplementary Table S3) who were diagnosed with isolated severe aortic stenosis and underwent aortic valve replacement surgery in the University Hospital Marqués de Valdecilla in Santander (Spain). Subepicardial biopsies (40–80 mg) were taken from the left ventricular lateral wall with a Tru-cut needle during the surgical procedure. Samples were all harvested by the same surgeon in a protocolized manner and always from the same location in the *margo obtusus* of the heart. Total RNA was obtained by TRIzol (Invitrogen) extraction. Real-time RT-PCR was conducted using specific TaqMan assays (Thermo Fisher) for the genes FOS and SIRT3.

This study followed the Declaration of Helsinki guidelines for biomedical research involving human subjects. Written informed consent was obtained from all patients prior to inclusion in the study.

### Statistical analysis

Statistical differences were established by either the Mann–Whitney test (in vivo experiments) and Student’s *t*-test for comparisons within two groups or ANOVA followed by Tukey’s test for multigroup comparisons using GraphPad Prism software.

## Supplementary information

Supplemental material
