# Supplementary material for: SIRT3-mediated inhibition of FOS through histone H3 deacetylation prevents cardiac fibrosis and inflammation
Source: Signal Transduct Target Ther. 2020 Feb 28;5:14. doi: 10.1038/s41392-020-0114-1 (PMC7046732; doi:10.1038/s41392-020-0114-1)
Supplement: Supplementary file 1 — Supplemental material [file 41392_2020_114_MOESM1_ESM.docx]

Supplementary Materials for

**SIRT3-mediated inhibition of FOS through histone H3 deacetylation prevents cardiac fibrosis and inflammation**

Xavier Palomer^1^, M. Silvia Román-Azcona^1^, Javier Pizarro-Delgado^1^, Ana Planavila^2^, Francesc Villarroya^2^, Brenda Valenzuela-Alcaraz^3^, Fátima Crispi^3^, Álvaro Sepúlveda-Martínez^3^, Irene Miguel-Escalada^4^, Jorge Ferrer^4,5^, J. Francisco Nistal^6^, Raquel García^7^, Mercy M. Davidson^8^, Emma Barroso^1^, Manuel Vázquez-Carrera^1,*^

^1^Department of Pharmacology, Toxicology and Therapeutic Chemistry, IBUB (Institut de Biomedicina de la Universitat de Barcelona), Research Institute - Pediatric Research Institute, Hospital Sant Joan de Déu, and CIBER de Diabetes y Enfermedades Metabólicas Asociadas (CIBERDEM), Faculty of Pharmacy and Food Sciences, University of Barcelona, Barcelona, Spain; ^2^Department of Biochemistry and Molecular Biomedicine, IBUB and CIBER Fisiopatología de la Obesidad y Nutrición (CIBEROBN), Faculty of Biology, University of Barcelona, Barcelona, Spain; ^3^aBCNatal - Barcelona Center for Maternal-Fetal and Neonatal Medicine (Hospital Clínic and Hospital Sant Joan de Deu), Institut d’Investigacions Biomèdiques August Pi i Sunyer, Universitat de Barcelona, and Center for Biomedical Research on Rare Diseases (CIBER-ER), Barcelona, Spain; ^4^Genomic Programming of Beta-cells Laboratory, Institut d’Investigacions August Pi i Sunyer (IDIBAPS), and CIBERDEM, Barcelona, Spain; ^5^Section of Epigenomics and Disease, Department of Medicine, and National Institute for Health Research (NIHR) Imperial Biomedical Research Centre, Imperial College London, London, United Kingdom; ^6^Servicio de Cirugía Cardiovascular, Hospital Universitario Marqués de Valdecilla, Departamento de Ciencias Médicas y Quirúrgicas, Facultad de Medicina, Universidad de Cantabria, Instituto de Investigación Marqués de Valdecilla (IDIVAL), Centro de Investigación Biomédica en Red Cardiovascular (CIBERCV), Instituto de Salud Carlos III, Santander, Spain; ^7^Departamento de Fisiología y Farmacología, Facultad de Medicina, Universidad de Cantabria, Instituto de Investigación Marqués de Valdecilla (IDIVAL), Santander, Spain; ^8^Department of Radiation Oncology, Columbia University, New York, NY, United States of America

Email addresses: Xavier Palomer ([xpalomer@ub.edu](mailto:xpalomer@ub.edu)), M. Silvia Román-Azcona ([silvita_rom@hotmail.com](mailto:silvita_rom@hotmail.com)), Javier Pizarro-Delgado ([jpizarro@ub.edu](mailto:jpizarro@ub.edu)), Ana Planavila ([aplanavila@ub.edu](mailto:aplanavila@ub.edu)), Francesc Villarroya ([fvillarroya@ub.edu](mailto:fvillarroya@ub.edu)), Brenda Valenzuela-Alcaraz ([valenzuela@clinic.cat](mailto:valenzuela@clinic.cat)), Fátima Crispi ([fcrispi@clinic.ub.es](mailto:fcrispi@clinic.ub.es)), Álvaro Sepúlveda-Martínez, Irene Miguel-Escalada ([miguel@clinic.cat](mailto:miguel@clinic.cat)), Jorge Ferrer, J. Francisco Nistal ([jfnistal@gmail.com](mailto:jfnistal@gmail.com)), Raquel García ([raquel.garcia@unican.es](mailto:raquel.garcia@unican.es)), Mercy M. Davidson ([mmd2@columbia.edu](mailto:mmd2@columbia.edu)), Emma Barroso ([ebarroso@ub.edu](mailto:ebarroso@ub.edu)), Manuel Vázquez-Carrera ([mvazquezcarrera@ub.edu](mailto:mvazquezcarrera@ub.edu)).

Correspondence to:  [Prof. Manuel Vázquez Carrera; Department of Pharmacology, Toxicology and Therapeutic Chemistry, Faculty of Pharmacy, University of Barcelona, Diagonal 643, E-08028, Barcelona, Spain. Tel: +34 934024531; Fax: +34 934035982. E-mail address: mvazquezcarrera@ub.edu](mailto:xxxxx@xxxx.xxx)

**This PDF file includes:**

Figures. S1 to S6

Tables S1 to S3


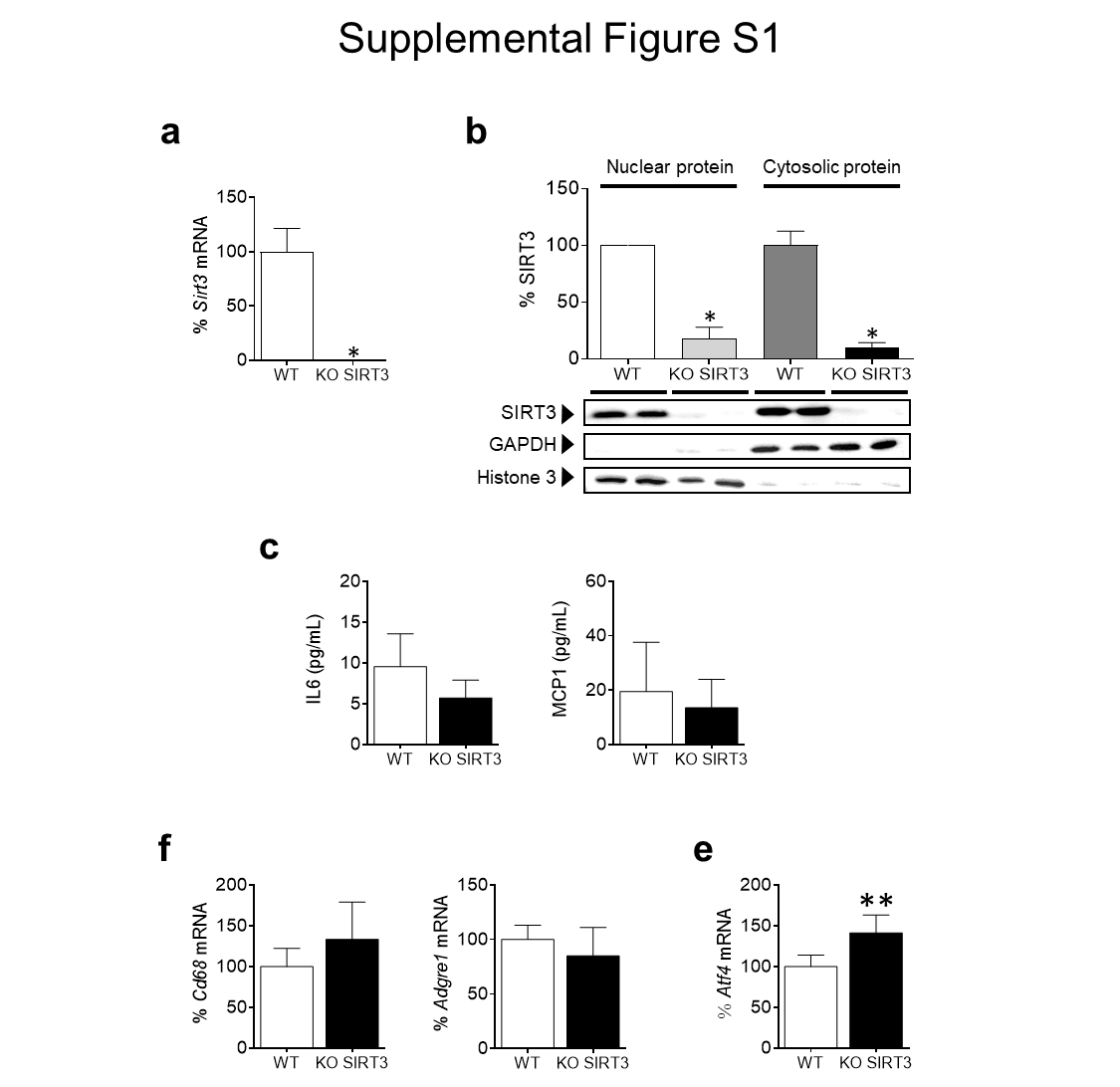
Figure. S1.

**Supplemental Fig. S1.** SIRT3 deletion does not induce systemic inflammation or increased macrophage infiltration in the heart. **a** Relative quantification of *SIRT3* mRNA expression in the heart of 7 to 9-month-old male SIRT3 knockout (KO SIRT3) mice and their wild-type (WT) littermates. **b** Western-blot analysis showing the levels of SIRT3 in cytosolic and nuclear protein fractions obtained from the same samples. Graphs represent the quantification of protein levels normalized to GAPDH (cytosolic protein) or histone 3 (nuclear protein), expressed as a percentage of WT samples ±SD. **c** Determination by ELISA of plasma IL6 and MCP1 levels in the plasma of 7-month-old male KO SIRT3 and their WT littermates. Relative quantification of *Cd68* and *Adgre1* (**d**), and *ATF4* (**e**) mRNA expression in the same samples depicted in panel **a**. Graphs represent the quantification of *Aprt*-normalized mRNA levels, expressed as a percentage of WT samples ±SD. Data were compared by the Mann–Whitney test (n=5-6). **P*<0.05, ***P*<0.01 and ****P*<0.001 vs. WT.


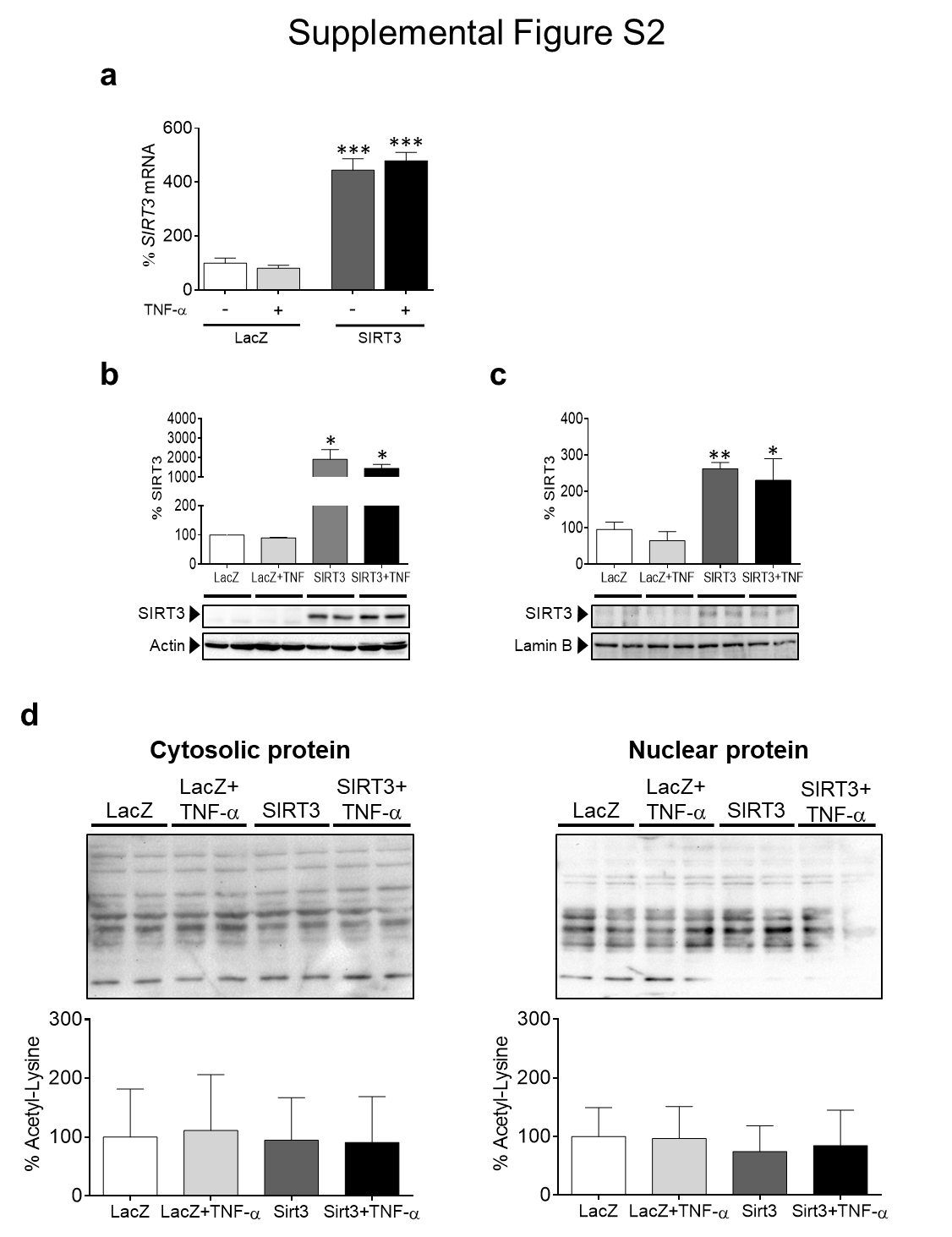
Figure. S2.

**Supplemental Fig. S2.** SIRT3 overexpression in human cardiac AC16 cells. **a** Relative quantification of *SIRT3* mRNA expression in human AC16 cardiac cells transfected with LacZ- or SIRT3-carrying plasmids in the presence or absence of TNF-α (TNF, 10 ng/mL, 24 h). Graphs represent the quantification of the glyceraldehyde-3-phosphate dehydrogenase (*GAPDH*)–normalized mRNA levels, expressed as a percentage of control samples ±SD. Western-blot analysis showing the levels of SIRT3 in total (**b**) and nuclear (**c**) protein fractions obtained from the same samples. Graphs represent the quantification of protein levels normalized to actin (total protein) or lamin B (nuclear protein), expressed as a percentage of control samples ±SD. (D) Western-blot analysis showing the levels of acetylated proteins in cytosolic and nuclear protein fractions obtained from the same samples depicted in panel **a**. Graphs represent the densitometric quantification of lysine acetylation normalized to actin or GAPDH (cytosolic protein), and lamin B (nuclear protein), expressed as a percentage of control LacZ samples ±SD (n=6-8, two independent AC16 cultures were used). Comparisons were performed by two-way ANOVA followed by Tukey post-test. **P*<0.05, ***P*<0.01 and ****P*<0.001 vs. LacZ; #*P*<0.05, ##*P*<0.01 and ###*P*<0.001 vs. LacZ+TNF-α.


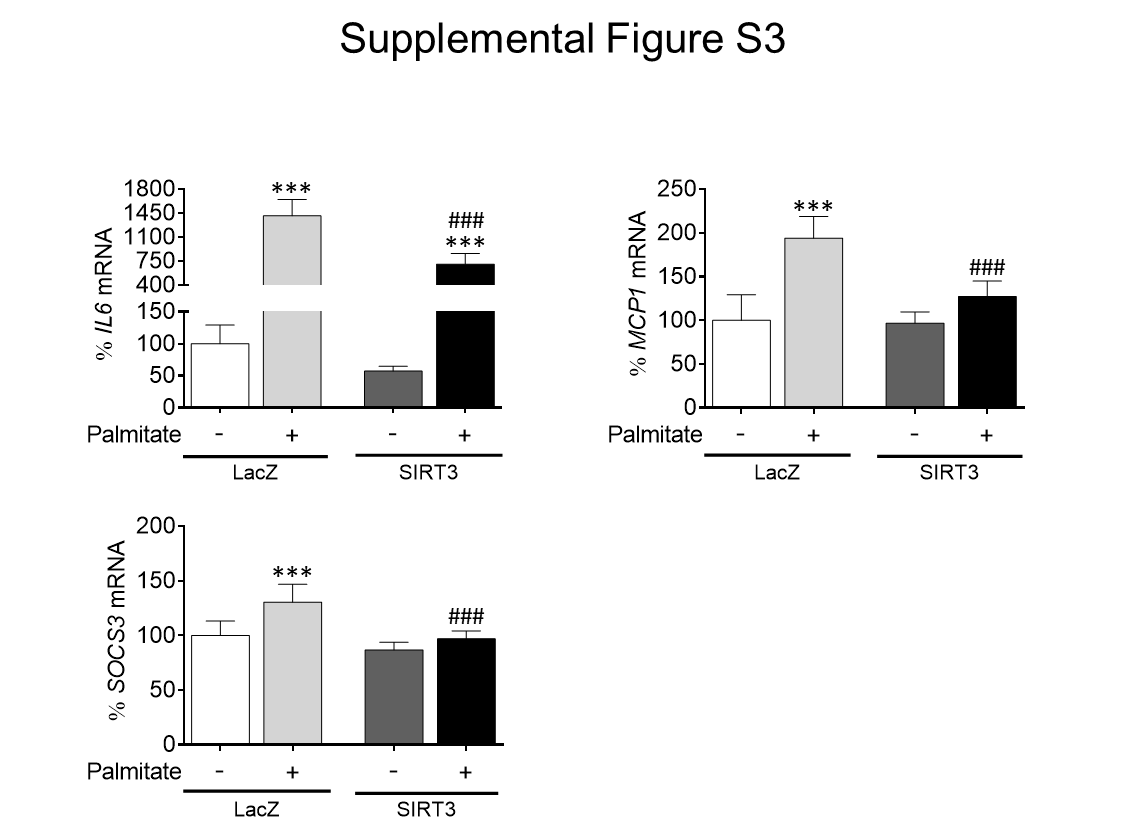
Figure. S3.

**Supplemental Fig. S3.** SIRT3 overexpression attenuates palmitate-induced inflammation in human cardiac cells. Relative quantification of *IL6*, *MCP1* and *SOCS3* mRNA expression in human AC16 cardiac cells transfected with LacZ- or SIRT3-carrying plasmids in the presence or absence of palmitate (0.25 mmol/L, 18h). Graphs represent the quantification of the *GAPDH*-normalized mRNA levels, expressed as a percentage of control samples ±SD (n=6). Comparisons were performed by ANOVA followed by Tukey post-test. **P*<0.05, ***P*<0.01 and ****P*<0.001 vs. LacZ; #*P*<0.05, ##*P*<0.01 and ###*P*<0.001 vs. LacZ+Palmitate.


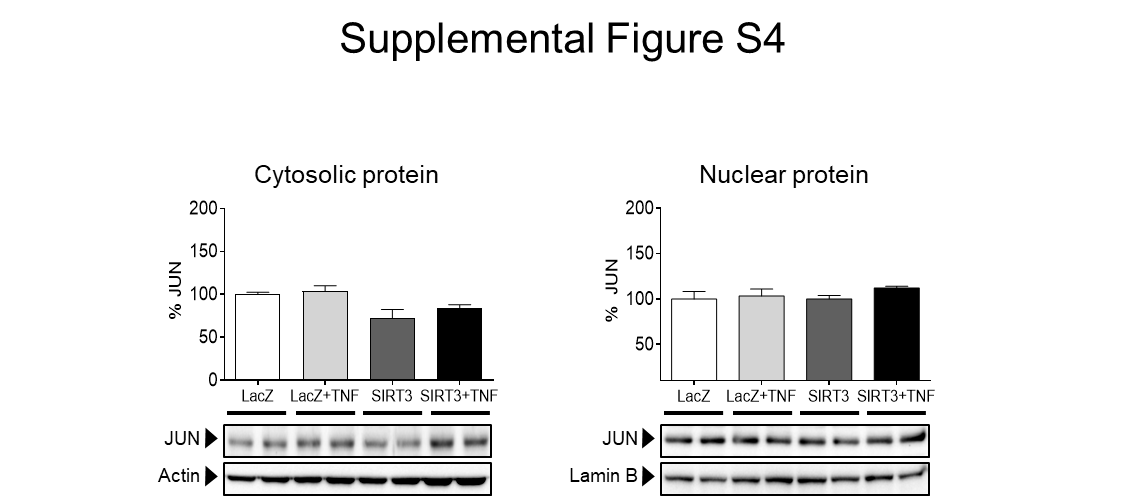
Figure. S4.

**Supplemental Fig. S4.** SIRT3 does not modulate JUN protein levels in human cardiac cells. Western-blot analysis showing JUN levels in cytosolic and nuclear protein fractions obtained from human AC16 cardiac cells transfected with LacZ- or SIRT3-carrying plasmids in the presence or absence of TNF-α (TNF, 10 ng/mL, 24h). Graphs represent the quantification of protein levels normalized to actin (cytosolic protein) or lamin B (nuclear protein), expressed as a percentage of control samples ±SD (n=4). Comparisons were performed by ANOVA followed by Tukey post-test. **P*<0.05, ***P*<0.01 and ****P*<0.001 vs. LacZ; #*P*<0.05, ##*P*<0.01 and ###*P*<0.001 vs. LacZ+TNF-α.


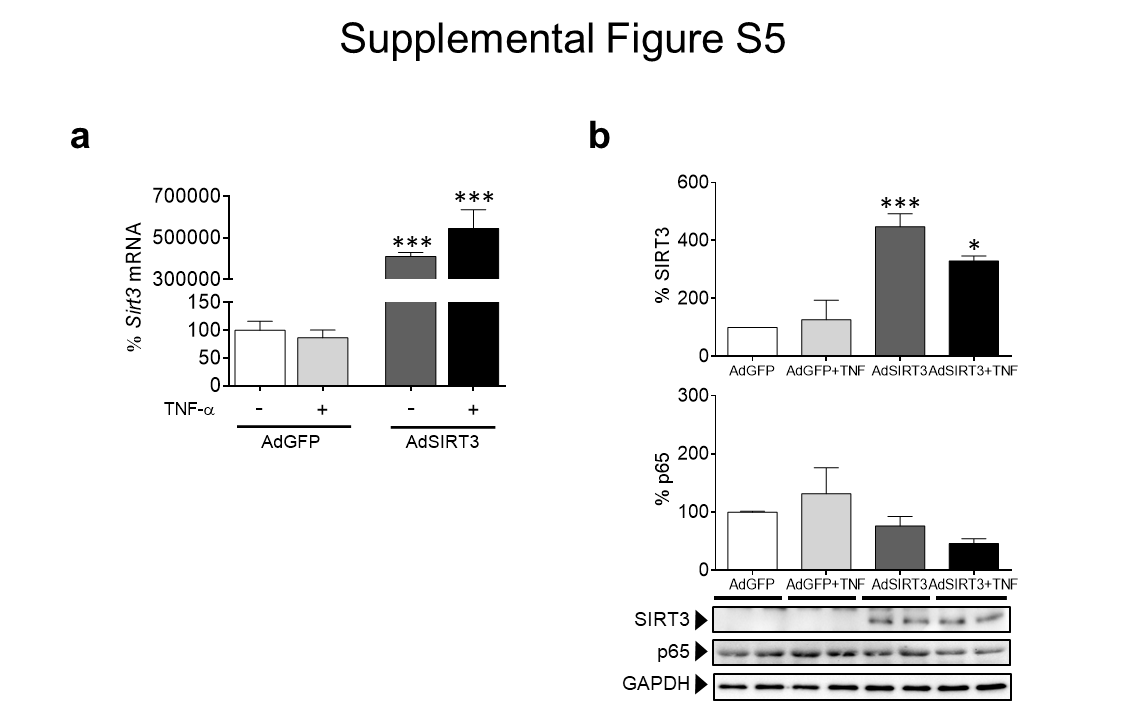
Figure. S5.

**Supplemental Fig. S5.** SIRT3 overexpression in neonatal rat cardiomyocytes. **a** Relative quantification of *Sirt3* mRNA expression in neonatal rat cardiomyocytes overexpressing Sirt3 (AdSIRT3) or GFP control vector (AdGFP; 30 IFU per cell, for 48h) in the presence or absence of TNF-α (TNF, 10 ng/mL, 24 h). Graph represents the quantification of the *GAPDH*–normalized mRNA levels, expressed as a percentage of control samples ±SD (n=5). **b** Western-blot analysis showing SIRT3 and p65 levels in total protein extracts obtained from neonatal rat cardiomyocytes overexpressing Sirt3 (AdSIRT3) or GFP control vector (AdGFP; 30 IFU per cell, for 48h) in the presence or absence of TNF-α (TNF, 10 ng/mL, 24 h). Graphs represent the quantification of protein levels normalized to GAPDH, expressed as a percentage of control samples ±SD (n=4). Data were compared by ANOVA followed by Tukey post-test. **P*<0.05, ***P*<0.01 and ****P*<0.001 vs. AdGFP; #*P*<0.05, ##*P*<0.01 and ###*P*<0.001 vs. AdGFP+TNF-α.

Figure. S6.

**
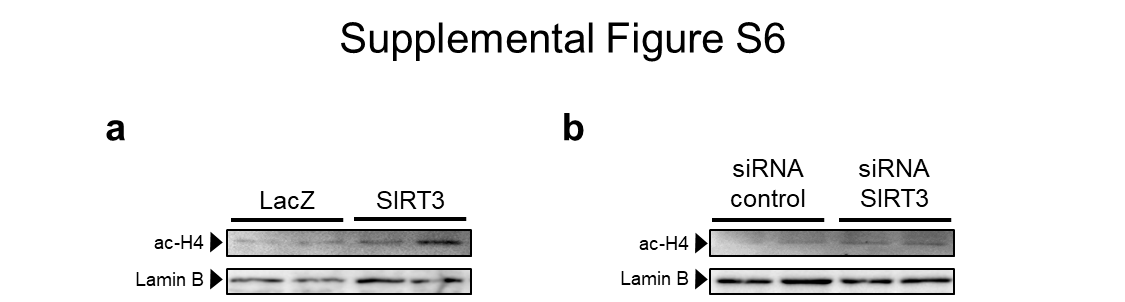
**

**Supplemental Fig. S6.** Western-blot analysis showing protein levels of acetylated histone H4 in nuclear protein extracts obtained from human AC16 cardiac cells transfected with LacZ- or SIRT3-carrying plasmids (**a**), or with scrambled siRNA or SIRT3 siRNA (**b**). Data were compared by ANOVA followed by Tukey post-test (n=4, two independent AC16 cultures were used).

Table S1. Echocardiographic parameters collected after ultrasound evaluation of mice.

|  | Group  Reverse primers | |  |
| --- | --- | --- | --- |
| Parameter | Wild type (WT) | SIRT3 knockout (KO) | *P* vs. WT |
| EF (%) | 77 (74-79) | 73 (70-76) | 0.110 |
| FS (%) | 40 (38-42) | 37 (34-40) | 0.138 |
| IVSd (mm) | 0.80 (0.77-0.87) | 0.82 (0.77-0.87) | 0.539 |
| IVSs (mm) | 0.85 (0.81-0.86) | 0.84 (0.79-0.89) | 0.902 |
| PWTd (mm) | 0.80 (0.69-0.89) | 0.69 (0.52-0.85) | 0.327 |
| PWTs (mm) | 0.84 (0.74-0.90) | 0.92 (0.80-1.00) | 0.140 |
| LV EDD (mm) | 2.87 (2.16-3.45) | 1.90 (1.71-2.07) | 0.085 |
| LV ESD (mm) | 2.71 (2.21-3.13) | 1.71 (0.90-2.32) | 0.065 |
| Left E (cm/sec) | 0.65 (0.59-0.69) | 0.65 (0.54-0.74) | 1.000 |
| MAPSE (mm) | 0.61 (5.9-6.1) | 0.59 (5.7-6.0) | 0.133 |
| Ao VTI (mm^2^) | 6.0 (5.6-6.4) | 5.7 (4.6-6.7) | 0.624 |
| Ao PV (mm/sec) | 0.95 (0.81-1.14) | 0.99 (0.92-1.05) | 0.462 |
| Heart rate (bpm) | 398 (309-480) | 391 (351-439) | 0.462 |
| TDI SEPT E (E’; cm/sec) | 0.06 (0.05-0.06) | 0.05 (0.04-0.05) | 0.260 |
| IVRT (msec) | 26 (22-30) | 22 (18-26) | 0.110 |
| Ratio E/E’ | 11.2 (10.4-11.9) | 12.1 (9.1-14.3) | 0.624 |

Data indicate median and interquartile range.

Abbreviations: EF, ejection fraction; FS, fractional shortening; IVSd, interventricular septal wall thickness at end-diastole; IVSs, interventricular septal wall thickness at end-systole; PWTd, left ventricular posterior wall thickness at end-diastole; PWTs, left ventricular posterior wall thickness at end-systole; LV EDD, left ventricular end-diastolic diameter; LV ESD, left ventricular end-systolic diameter; Left E, early transvalvular filling; MAPSE, mitral annular plane systolic excursion; Ao VTI, aortic velocity time integral (aortic valve area); Ao PV, aorta peak velocity; TDI SEPT E, early diastolic annular peak velocity; IRT, isovolumetric relaxation time.

Table S2. Primers used for the real-time RT-PCR reactions.

| Gene | Forward primers | Reverse primers |
| --- | --- | --- |
| **Human** |  |  |
| *ATF4* | 5’-GCGGGCTCCTCCGAAT-3’ | 5’-ATCCTCCTTGCTGTTGTTGGA-3’ |
| *FOS* | 5’-GGGATAGCCTCTCTTACTACCACTCA-3’ | 5’-GAAGTCCTGCGCGTTGACA-3’ |
| *EDN-1* | 5’-TGCCACCTGGACATCATTTG-3’ | 5’-TGGACCTAGGGCTTCCAAGTC-3’ |
| *GAPDH* | 5’-GGCCTCCAAGGAGTAAGACC-3’ | 5’-AGGGGTCTACATGGCAACTG-3’ |
| *ICAM1* | 5’- GCCAGGAGACACTGCAGACA-3’ | 5’- TGGCTTCGTCAGAATCACGTT-3’ |
| *IL6* | 5’-CCCCCAGGAGAAGATTCCAA-3’ | 5’-TCAATTCGTTCTGAAGAGGTGAGT-3’ |
| *MCP1* | 5’-GCTGTGATCTTCAAGACCATTGTG-3’ | 5’-TGGAATCCTGAACCCACTTCTG-3’ |
| *SIRT3* | 5’-CGGCTCTACACGCAGAACATC-3’ | 5’-AGGTTCCATGAGCTTCAACCA-3’ |
| *SOCS3* | 5’-GACCAGCGCCACTTCTTCAC-3’ | 5’-CTGGATGCGCAGGTTCTTG-3’ |
| *TGFB1* | 5’-AACCCACAACGAAATCTATGAC-3’ | 5’-GAGGTATCGCCAGGAATTGT-3’ |
| *TNF-α* | 5’-TCTTCTCGAACCCCGAGTGA-3’ | 5’-GGAGCTGCCCCTCAGCTT-3’ |
| **Mice** |  |  |
| *Aprt* | 5’-CAGCGGCAAGATCGACTACA-3’ | 5’-AGCTAGGGAAGGGCCAAACA-3’ |
| *Adgre1* | 5’-AAGGCCCAGGAGTGGAATGT-3’ | 5’-GGATATTGGTGCAGACTGAGTTAGG-3’ |
| *Atf4* | 5’-AGCAAAACAAGACAGCAGCC-3’ | 5’-ACTCTCTTCTTCCCCCTTGC-3’ |
| *β-MHC* | 5’-GGAATGTTAGAGATATTTTTGCTTCACTTT-3’ | 5’-AAACAATTGGAAGTGGTCGTCAT-3’ |
| *Cd68* | 5’-GGACTACATGGCGGTGGAATA-3’ | 5’-GATGAATTCTGCGCCATGAA-3’ |
| *Fos* | 5’-GAGGAGGGAGCTGACAGATACACT-3’ | 5’-GATTGGCAATCTCAGTCTGCAA-3’ |
| *Icam1* | 5’-GTCCGCTGTGCTTTGAGAACT-3’ | 5’-CGGAAACGAATACACGGTGAT-3’ |
| *Il6* | 5’-ACACATGTTCTCTGGGAAATCGT-3’ | 5’-AAGTGCATCATCGTTGTTCATACA-3’ |
| *Mcp1* | 5’-GCTGGAGAGCTACAAGAGGATCA-3’ | 5’-CTCTCTCTTGAGCTTGGTGACAAA-3’ |
| *Nppb* | 5’-GCCAGTCTCCAGAGCAATTCA-3’ | 5’-GGGCCATTTCCTCCGACTT-3’ |
| *Socs3* | 5’-ATGGTCACCCACAGCAAGTTT-3’ | 5’-TCCAGTAGAATCCGCTCTCCT-3’ |
| *Sod2* | 5’-CAGGACCCATTGCAAGGAA-3’ | 5’-GTGCTCCCACACGTCAATCC-3’ |
| *Tnf-α* | 5’-AGCCGATGGGTTGTACCTTGT-3’ | 5’-TGAGATAGCAAATCGGCTGAC-3’ |
| **Rat** |  |  |
| *Aprt* | 5’-CAGCGGCAAGATCGACTACA-3’ | 5’-AGCTAGGGAAGGGCCAAACA-3’ |
| *Edn-1* | 5’-TGATTCTCTTGCCTCTTCTTG-3’ | 5’-TATGGAATCTCCTGGCTCTC-3’ |
| *Fos* | 5’-GACAGCCTTTCCTACTACCATTCC-3’ | 5’-CGCAAAAGTCCTGTGTGTTGA-3’ |
| *Icam1* | 5’-GGCTGTCACTGTTCAAGAATGTCT-3’ | 5’-TCAGGACCCTAGTCGGAAGATC-3’ |
| *Il6* | 5’-ATATGTTCTCAGGGAGATCTTGGAA-3’ | 5’-GTGCATCATCGCTGTTCATACA-3’ |
| *Mcp1* | 5’-CTGTCTCAGCCAGATGCAGTTAA-3’ | 5’-TGGGATCATCTTGCCAGTGA-3’ |
| *Tgfb1* | 5’-AAGAAGTCACCCGCGTGCTA-3’ | 5’-TGTGTGATGTCTTTGGTTTTGTCA-3’ |
| *Tnf-α* | 5’-GACCCTCACACTCAGATCATCTTCT-3’ | 5’-TCCGCTTGGTGGTTTGCTA-3’ |

Abbreviations: APRT, adenine phosphoribosyl transferase; ADGRE1, adhesion G protein-coupled receptor E1; ATF4, activating transcription factor 4; β-MHC, β-myosin heavy chain; EDN-1, endothelin 1; FOS, Fos proto-oncogene, AP-1 transcription factor subunit; GAPDH, glyceraldehyde-3-phosphate dehydrogenase; ICAM1, intercellular adhesion molecule 1; IL6, interleukin 6; MCP1, monocyte chemoattractant protein 1; NPPB, natriuretic peptide type B; SIRT3, sirtuin 3; SOCS3, suppressor of cytokine signaling 3; SOD2, superoxide dismutase 2; TGFB1, transforming growth factor β; TNF-α, tumor necrosis factor α.

Table S3. Study population characteristics.

| Total number of subjects | 26 |
| --- | --- |
| Male | 26 |
| Mean age (mean ± SD) | 64.6 ± 13.9 |
| Hypertension | 14 (54%) |
| Diabetes mellitus | 10 (38%) |
| Hyperlipidemia | 8 (31%) |
| Obesity (BMI ≥30 kg/m^2^) | 5 (19%) |
| Smoker  Ex-smoker | 6 (23%)  7 (27%) |
| Sinus rythm | 22 (85%) |
| Atrial fibrillation | 2 (8%) |
| Permanent pacemaker | 2 (8%) |
| Mean LV ejection fraction (%, mean ± SD) | 57.1 ± 14.2 |
| Mean indexed LVM (g/m^2.7^, mean ± SD)  Subjects with iLVM (< 51 g/m^2.7^) | 72.7 ± 18.5  4 (15%) |

Abbreviations: iLVM, inappropriate LVM; LV, left ventricle; LVM, LV mass.
